# Generation of Combinatorial Lentiviral Vectors Expressing Multiple Anti-Hepatitis C Virus shRNAs and Their Validation on a Novel HCV Replicon Double Reporter Cell Line

**DOI:** 10.3390/v12091044

**Published:** 2020-09-18

**Authors:** Hossein M. Elbadawy, Mohi I. Mohammed Abdul, Naif Aljuhani, Adriana Vitiello, Francesco Ciccarese, Mohamed A. Shaker, Heba M. Eltahir, Giorgio Palù, Veronica Di Antonio, Hanieh Ghassabian, Claudia Del Vecchio, Cristiano Salata, Elisa Franchin, Eleonora Ponterio, Saleh Bahashwan, Khaled Thabet, Mekky M. Abouzied, Ahmed M. Shehata, Cristina Parolin, Arianna Calistri, Gualtiero Alvisi

**Affiliations:** 1 Department of Pharmacology and Toxicology, College of Pharmacy, Taibah University, Almadinah Almunawwarah 41477, Saudi Arabia; hossein.elbadawy@hotmail.com (H.M.E.); njohany@taibahu.edu.sa (N.A.); heba_m.eltahir@yahoo.com (H.M.E.); salehbahashwan@gmail.com (S.B.); m_meky2001@yahoo.com (M.M.A.); ahmedshehata73@yahoo.co.uk (A.M.S.); 2 Department of Molecular Medicine, University of Padua, 35121 Padua, Italy; adriana.vitiello89@gmail.com (A.V.); fran.ciccarese@gmail.com (F.C.); giorgio.palu@unipd.it (G.P.); veronicadiantonio@libero.it (V.D.A.); haniehghassabian@gmail.com (H.G.); claudia.delvecchio@unipd.it (C.D.V.); cristiano.salata@unipd.it (C.S.); elisa.franchin@unipd.it (E.F.); eleonoraponterio@yahoo.it (E.P.); cristina.parolin@unipd.it (C.P.); 3 Immunology and Molecular Oncology Unit, Veneto Institute of Oncology IOV-IRCCS, 35128 Padua, Italy; 4 Pharmaceutics and Pharmaceutical Technology Department, College of Pharmacy, Taibah University, Almadinah Almunawwarah 41477, Saudi Arabia; shaker_pharmacy2002@yahoo.com; 5 Pharmaceutics Department, Faculty of Pharmacy, Helwan University, Cairo 11795, Egypt; 6 Fondazione Policlinico Universitario "A. Gemelli"—I.R.C.C.S., 00168 Rome, Italy; 7 Department of Biochemistry, Faculty of Pharmacy, Minia University, Minia 61519, Egypt; Khaled_thabet@minia.edu.eg; 8 Department of Pharmacology and toxicology, Faculty of Pharmacy, Beni-Suef University, Beni-Suef 62511, Egypt

**Keywords:** hepatitis C virus, reporter cell line, antivirals, gene therapy, siRNA

## Abstract

Despite the introduction of directly acting antivirals (DAAs), for the treatment of hepatitis C virus (HCV) infection, their cost, patient compliance, and viral resistance are still important issues to be considered. Here, we describe the generation of a novel JFH1-based HCV subgenomic replicon double reporter cell line suitable for testing different antiviral drugs and therapeutic interventions. This cells line allowed a rapid and accurate quantification of cell growth/viability and HCV RNA replication, thus discriminating specific from unspecific antiviral effects caused by DAAs or cytotoxic compounds, respectively. By correlating cell number and virus replication, we could confirm the inhibitory effect on the latter of cell over confluency and characterize an array of lentiviral vectors expressing single, double, or triple cassettes containing different combinations of short hairpin (sh)RNAs, targeting both highly conserved viral genome sequences and cellular factors crucial for HCV replication. While all vectors were effective in reducing HCV replication, the ones targeting viral sequences displayed a stronger antiviral effect, without significant cytopathic effects. Such combinatorial platforms as well as the developed double reporter cell line might find application both in setting-up anti-HCV gene therapy approaches and in studies aimed at further dissecting the viral biology/pathogenesis of infection.

## 1. Introduction

The recent introduction of hepatitis C virus (HCV) NS3, NS5B, and NS5A inhibitors, the so-called directly acting antivirals (DAAs), has greatly improved HCV treatment [1]. However, due to the biological characteristics of HCV, the emergence of drug-resistance represents an important limitation. Furthermore, high cost and lack of patient compliance constitute additional problems [2]. Thus, the design of innovative therapeutic strategies and the development of additional agents to fight HCV are worth being considered.

Silencing of specific genes can be achieved by RNA interference (RNAi) that is based on the degradation of target mRNAs by small complementary RNA molecules known as small interfering RNAs (siRNAs) [3,4]. HCV is highly susceptible to RNAi, because its genome is a positive single-stranded RNA, which is involved both in viral transcription and cytoplasmic replication. Thus, RNAi might represent a promising therapeutic tool against HCV as targeting viral genome through specific siRNAs should halt viral replication and propagation. Furthermore, it has been previously demonstrated that not only silencing of viral factors, but also interfering with cellular proteins known to play a role in HCV life cycle, would block viral replication [5,6,7,8,9,10,11,12]. In particular, it has been shown that combinatorial strategies based on the co-expression of arrays of siRNAs targeting multiple viral and/or cellular genes displayed synergistic anti-HCV effects, hence reducing the possibility of resistant variant emergence [13,14,15,16].

Suitable levels of RNA transgenes expression are necessary for the success of this approach. In this respect, lentiviral vectors possess several properties which make them excellent gene delivery systems: (i) they integrate into dividing and non-dividing cells, (ii) they are able to transduce a significant percentage of cells in ex vivo culture and in vivo procedures, and (iii) a safer integration profile when compared to the one of gamma-retroviral vectors. HIV-1 derived vectors are currently being investigated for in vivo gene therapy and several clinical trials are ongoing; no severe adverse events have been reported so far (http://www.abedia.com/wiley/vectors.php, accessed on 1^st^ May 2020).

As mentioned above, given the genetic diversity of HCV that results from the high mutation rate during viral replication, designing siRNAs to target cellular components confers more stability and efficacy. Recently, a number of host factors, absolutely required for virus life cycle, have been identified. Among them, phosphatidylinositol-4 kinase III alpha (PI4KIIIα) that is involved in the formation of double membrane vesicles where RNA replication takes place [17,18]; cyclophilin A (CypA), a host chaperone whose prolyl-peptidyl isomerase activity is essential for incorporation of NS5B in the replication compartments [19,20,21,22]; and the liver specific miR-122, which binds to 5′ HCV UTR, thus stimulating viral polyprotein translation [23], emerged as extremely promising targets of antiviral therapy [24,25,26]. Indeed, both the CypA inhibitor alisporivir and the miR-122 antisense RNA miravirsen have already been studied in clinical trials [27,28], whereas specific PI4KIIIα inhibitors have been proposed as ideal candidates for the development of a new class of anti-HCV drugs [29].

As far as the viral transcripts are concerned, one needs to keep in mind that seven major HCV genotypes and numerous subtypes have been described, and that the genotype nucleotide sequences differ by as much as 30% [30]. In addition, due to the biological characteristics of the viral RNA-dependent RNA polymerase (NS5B), HCV RNA genome incorporates mutations at each replication cycle, resulting in the emergence of genetically distinct, but closely related, variants known as “quasispecies”. Thus, when selecting HCV genomic regions to be targeted by siRNAs, the ones with the highest degree of conservation must be identified and carefully chosen. HCV genome is a (+) single-stranded RNA molecule of 9.6 kb bearing a single open reading frame (ORF) flanked at the 5′ and 3′ ends by highly conserved and structured non-translated regions (NTRs), involved in viral translation and replication [31]. The NTR mapping at the 5′ end is composed of four secondary structured domains (I, II, III, and IV) and contains an internal ribosome entry site (IRES) which is essential for cap-independent translation of the viral RNA. As mentioned above, this region is highly conserved among different HCV isolates and thus represents an interesting target for therapeutic interventions.

In a previous study, siRNAs targeting highly accessible and conserved sequences within the HCV IRES were identified by employing an in silico design, followed by a selection protocol complemented by an automated MysiRNA-Designer pipeline. Importantly, off-target effects were excluded by appropriate analysis. In particular, two siRNAs (HCV353 and HCV321 targeting extremely well conserved sequences, respectively in stem loop IV and stem loops III/IV of HCV IRES) were characterized by a predicted high specificity and antiviral efficacy. Indeed, HCV353 inhibited RNA of several clinical isolates at low concentrations and after short exposure times. Intriguingly, no emergence of escape mutant viruses was found after prolonged treatment with HCV353 [32].

We and others have previously shown that platforms simultaneously expressing multiple short harpin RNAs (shRNAs) can be developed by adopting one pol-III promoter to express long harpin RNAs (lhRNAs) or extended shRNAs, or by generating arrays of pol-III/shRNA units [33]. Here, we generated lentiviral vectors expressing different combination of selected shRNAs already known to interfere with HCV replication cycle and we compared them for their efficacy in terms of therapeutic molecule expression and anti-viral activity. To this end, we developed a novel HCV subgenomic replicon double reporter cell line that might find additional application in setting up and testing innovative therapeutic interventions and molecules to tackle HCV replication/infection.

## 2. Materials and Methods

### 2.1. Compounds

Sofosbuvir (PSI-7977) was purchased from MedChemTronica (Bergkällavägen, Sweden). AL-9 [29] was a generous gift from Raffaele DeFrancesco (INGM, Milan, Italy). B3 was purchased from Vitas-M Laboratories (Causeway Bay, Hong Kong, China). All compounds were resuspended in dimethyl sulfoxide (DMSO) and stored at −20 °C in the dark until needed.

### 2.2. Plasmids

Plasmid RLuc-UL44, mediating expression of a N-terminal fusion of Renilla Luciferase (RLuc) to human cytomegalovirus DNA polymerase processivity factor UL44 [34], was used as template for amplification with primers FW-RLuc (5′-GGGGACAAGTTTGTACAAAAAAGCAGGCTTGACCAGCAAGGTGTACGAC-3′) and RV-RLuc (5′-GGGGACCACTTTGTACAAGAAAGCTGGGTTTAGAGATCCCCCTGCTCGTTC-3′) to generate a PCR product containing the RLuc coding sequence flanked by Gateway compatible attB sites. The purified PCR product was used with plasmid pDNR207 (Thermofisher Scientific, Waltham, MA, USA) for a Gateway BP recombination reaction to generate entry clone pDNR-RLuc, as described in [35]. Entry clone pDNR-RLuc was used with the lentiviral Expression vector pWPI_nHA_puro_rfB for Gateway LR recombination reactions as described previously [36], to generate Lentiviral expression vector pWPI_nHA_RLuc_puro, mediating expression of N-terminally HA-tagged RLuc under the control of the EF1α promoter [37], Packaging plasmid pCMVΔ8.74 (Addgene #22036) and the VSV envelope glycoprotein expression vector pMD2.G (Addgene #12259) were kindly provided by Didier Trono (Lausanne, Switzerland). Selected shRNAs were inserted between the XbaI and XhoI sites of the third generation self-inactivating lentiviral vector pLL3.7 under the transcriptional control of different pol-III promoters (Bio-fab research, Rome, Italy). The predicted folding of each shRNA or lhRNA was calculated by adopting Mfold Web Server setting default parameters at 37 °C [38].

### 2.3. Cell Lines

Human embryonic kidney (HEK) 293T (ATCC^®^ CRL-3216™), Huh7.5 (kindly provided by Charlie Rice, The Rockefeller University, New York, NY, USA), and Huh7-Lunet cells (a generous gift from Ralf Bartenschlager, University of Heidelberg, Heidelberg, Germany) were maintained in Dulbecco’s modified Eagle’s medium (DMEM, Thermo Fischer Scientific, Waltham, MA, USA) supplemented with 10% (*v*/*v*) fetal bovine serum (FBS), 50 U/mL penicillin, 50 U/mL streptomycin, and 2 mM L-glutamine, as described previously [39]. The Huh7-Lunet cell line, allowing efficient replication of the JFH1 HCV genome [40], was used to generate the Huh7-Lunet-RLuc cell line by lentiviral transduction. For production of lentiviral particles, 1.2 × 10^6^ HEK293T cells were seeded into 6 cm-diameter dishes and transfected using the CalPhos mammalian transfection kit (Becton Dickinson Franklin Lakes, NJ, USA), as described previously [41]. Briefly, 30 min prior to transfection, the medium was replaced. For transfection, 6.4 μg packaging plasmid (pCMVΔ8.91), 6.4 μg of pWPI-HA-RLuc and 2.1 μg of the VSV envelope glycoprotein expression vector (pMD2.G) were mixed and diluted to a final volume of 438 μL in H_2_O. Then, 62 μL 2M CaCl_2_ and 500 μL HEPES Buffered Saline precipitation buffer 2× (HBS 2×) were added. The mixture was immediately added to the cell culture dish in a drop-wise fashion and the plate was gently swirled to evenly distribute the transfection mixture throughout the plate. After 8 h, medium was replaced by 5 mL of fresh medium. On the next day, 8 × 10^4^ Lunet cells were seeded into a 10 cm-diameter dish; 24 h later (48 h post-transfection), the supernatant containing lentiviral particles was harvested and replaced by 4 mL of fresh DMEM. Supernatants were filtered through a 0.45 μm-pore membrane prior to usage. Transduction of target cells with the lentiviral particles was repeated three times every 12 h to achieve high number of integrates and thus high expression levels. Transduced cell pools were subjected to selection with medium containing 1 μg/mL Puromycin, as described previously [42].

### 2.4. In Vitro Transcription

Briefly, 10 μg of pFKI389Luc-ubi-neo/NS3-3′_dg_JFH plasmid were linearized by restriction with MluI and purified using the Nucleospin Extract II kit (Macherey-Nagel, Düren, Germany) as described previously [43]. In vitro transcription reaction mixtures contained 80 mM HEPES (pH 7.5), 12 mM MgCl_2_, 2 mM spermidine, 40 mM dithiothreitol (DTT), 3.125 mM of each nucleoside triphosphate, 100 U of RNasin (Promega, Madison, WI, USA), 10 μg plasmid DNA, and 80 U of T7 RNA polymerase (Promega, Madison, WI, USA) in 100 μL final volume. After incubation for 2 h at 37 °C, 40 U of T7 RNA polymerase were added and the mixture was incubated for further 2 h at 37 °C. RNA was extracted with acidic phenol and chloroform, precipitated with isopropanol, and dissolved in RNase-free water. RNA integrity was determined by using denaturing agarose gel electrophoresis and concentration was determined by measuring optical density at 260 nm.

### 2.5. Generation of HCV Replicon Cell Lines

The FLuc-JFH1/RLuc stable replicon cell line was generated as described previously [44]. To this end, a single-cell suspension of 6 × 10^4^ Huh7-Lunet/RLuc cells was prepared by trypsinization and washed once with phosphate-buffered saline (PBS). Cells were resuspended in 400 μL of Cytomix [45] containing 2 mM ATP and 5 mM glutathione and mixed with 1 μg of in vitro transcribed pFKI389Luc-ubi-neo/NS3-3′_dg_JFH RNA before being transfected by electroporation with a Gene Pulser system (Bio-Rad, Hercules, CA, USA) in a cuvette with a gap width of 0.4 cm (Bio-Rad, Hercules, CA, USA) at 975 μF and 270 V, as described previously [41]. Cells were plated in a T75 Flask and, three days post-electroporation, G418 was added to a final concentration of 750 μg/mL in order to allow selection of a mixed cell population stably replicating HCV genome.

### 2.6. MTT Cell Viability Assay

Huh7.5 cells were seeded in 96-well plates (9 × 10^4^ cells/cm^2^) and 24 h later were transduced with the lentiviral particles described before, at the Multiplicity Of Infection (MOI) of 0.062 TU/cell. At 48 and 72 h p.t., cells were processed for 3-(4,5-Dimethylthiazol-2-yl)-2,5-Diphenyltetrazolium Bromide (MTT) assay (Roche, Basel, Switzerland), according to the manufacturer’s specifications.

### 2.7. Quantification of HCV Replication

FLuc-JFH1/RLuc or Lunet cells were seeded in 24-well plates. At the desired time point, cells were washed with 1 mL PBS/well and lysed in 100 μL/well of Passive Lysis Buffer 1× (PLB 1×, Promega, Madison, WI, USA). Plates were shaken 15 min at 500 rpm and freeze/thawed. Subsequently, 75 μL from each well were transferred to 96-well solid black flat bottom polystyrene TC-treated microplates (Costar^®^, Washington, DC, USA). Fluorescent GFP signals, proportional to transduction efficiency with pRRL3 silencing lentiviral vectors, were acquired for 1 s/well using a spectrometer compatible with fluorescent measurements (VICTOR X2 Multilabel Plate Reader, PerkinElmer, Waltham, MA, USA) in combination with a fluorometric excitation filter (band pass 485 ± 14 nm) and a fluorometric emission filter (band pass 535 ± 25 nm). Before reading, the plate was shaken for 1 s at normal speed and with double orbit. After background subtraction using values relative to untransduced cells, the data obtained were used to calculate the GFP signal to each condition. FLuc counts, proportional to HCV replication, were acquired by transferring 75 µL/well of cell lysates to 96-well solid white flat bottom polystyrene TC-treated microplates (Costar^®^, Washington, DC, USA, product number 3916), followed by addition of 75 µL/well of Dual-Glo^®^ Luciferase assay reagent (Promega, Madison, WI, USA) and by integrating luminescent signals for 30 min. RLuc counts, proportional to cell number, were finally acquired by adding 75 µL/well of Dual-Glo^®^ Stop&Glo reagent (Promega, Madison, WI, USA) to each sample in order to quench FLuc signals. Data were analysed and plotted with Excel (Microsoft, Redmond, Washington, United States) and GraphPad Prism 6 (GraphPad software, San Diego, CA, USA).

### 2.8. Statistical Analysis

We compared the effect of transduction with the different Lentiviral particles using one-way analysis of variance (ANOVA) and Tukey’s multiple-comparison post-test to calculate multiplicity adjusted *p* values for each comparison. Differences between groups were considered to be significant at a *p* value of <0.05. Statistical analyses were performed with GraphPad Prism 6 (GraphPad Software, Inc., San Diego, CA, USA).

### 2.9. Cell Treatment with Antiviral Drugs

FLuc-JFH1/RLuc or Lunet cells were seeded in 24-well plates (2.5 × 10^4^ cells/cm^2^) and 24 h later were treated with increasing concentrations of either Sofosbuvir, AL-9, B3 or DMSO. 72 h post-treatment, cells were processed for quantification of HCV replication as described above.

### 2.10. Production and Titration of Lentiviral Particles

For production of lentiviral particles, 2.5 × 10^6^ HEK293T cells were seeded into 10 cm-diameter dishes and transfected as described above. After filtration through a 0.45 µm-pore-size membrane, lentiviral particle containing cell supernatants were concentrated 10× using Vivaspin 20, 100,000 MWCO PES (Sartorius, Goettingen, Germany) and aliquots stored at −80 °C until needed.

For titration, 5 × 10^4^ HEK293T or 4 × 10^4^ Huh7-Lunet cells/well were seeded in 24-multi well plates, and 24 h later, cells were transduced with serial dilutions of lentiviral stocks and incubated for 72 h. Subsequently, cells were washed three times with PBS and the GFP expression was measured by adopting FACS Calibur (Becton Dickinson Franklin Lakes, NJ, USA), as previously described [33].

### 2.11. Quantification of Anti-HCV shRNA/lhRNAs Expression by Real Time PCR

The expression levels of the selected shRNAs/lhRNAs were assessed in transduced Huh7.5 cells (2.1 × 10^4^ cells/cm^2^ transduced a MOI of 1.25 TU/cell for each lentivirus). Seventy-two h p.t., RNA was extracted and a Real Time PCR assay was performed using the Chen’s stem–loop RT-PCR method [46]. Briefly, a set of oligonucleotides for the 4 selected shRNas was designed, along with specific probes. Subsequently, the system was tested on plasmid DNA containing the respective shRNA encoding sequence. Once assessed the performance of the designed method, total RNA was extracted from transduced Huh7.5 cells by adopting the mirVana™ miRNA Isolation Kit with phenol (Thermo Fischer Scientific, Waltham, MA, USA) and was retro-transcribed and amplified by the TaqMan^®^ Small RNA Assays (Applied Biosystem, Foster City, CA, USA) method, following the manufacturer’s instruction.

### 2.12. Western Blotting

FLuc-JFH1/RLuc cells were seeded in 6-well plates (2.5 × 10^4^ cells/cm^2^) and 24 h later were treated with increasing concentrations of Sofosbuvir in DMSO 0.1% (*v*/*v*); 72 h later, cells were washed with ice-cold PBS and lysed in RIPA buffer with protease (cOmplete™ Protease Inhibitor Cocktail, Merck, Kenilworth, NJ, USA) and phosphatase (PhosSTOP, Merck, Kenilworth, NJ, USA) inhibitors. Cell lysates were gently recovered by scraping, further incubated for 15 min in ice, and soluble material recovered by centrifugation at 13,000 rpm for 30 min at 4 °C. 10 µL of each sample was analyzed by SDS page/Western blotting as described previously [47]. The following antibodies, diluted in PBS containing 0.2% Tween20 and 5% milk (*w*/*v*), were used: α-NS5 clone 9-E10 mouse mAb (a generous gift from Charles Rice, The Rockefeller University, New York, NY, USA; 1:10,000), α-αTubulin mouse mAb (T6074, Merck, Kenilworth, NJ, USA; 1:10,000); and goat α-mouse immunoglobulin Ab conjugated to horseradish peroxidase (#sc-2055, Santa Cruz Biotech, Dallas, TX, USA; 1:10,000). Signals were acquired using an imaging system (Alliance Mini, Uvitec Cambridge, UK) and quantified using Image J (NIH).

## 3. Results

### 3.1. Generation of Lentiviral Vectors Expressing Different Arrays of Anti-HCV shRNAs

**Figure 1:**
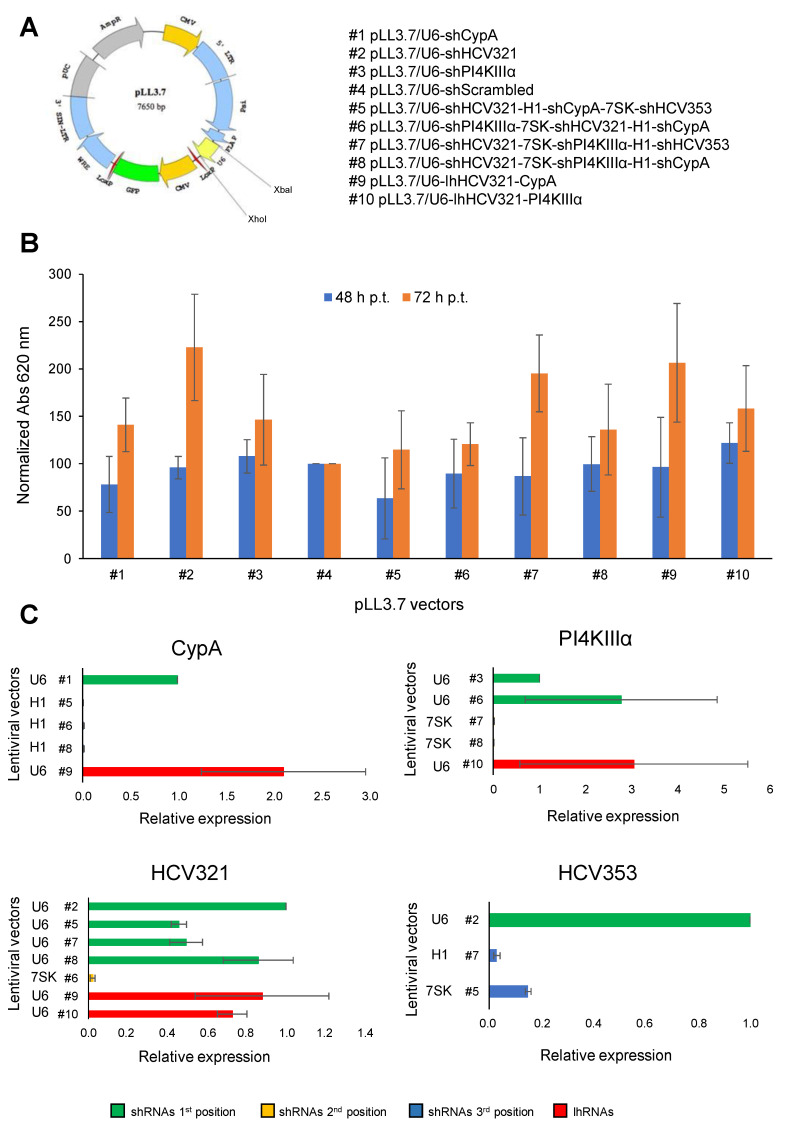
Generation and characterization of lentiviral particles encoding shRNAs targeting hepatitis C virus (HCV) replication. (**A**) Schematic representation of the different lentiviral vectors generated. The shRNA cassettes were inserted between the XbaI and XhoI sites of lentiviral vector pLL3.7 under the control of pol-III promoters. The name of the different vectors is reported on the right panel. (**B**) Effect of lentiviral transduction on cell growth and viability; 9 × 10^4^ Huh7.5 cells/cm^2^ were seeded in 96-well plates; 24 h later cells were transduced with the lentiviral particles described in panel (**A**), at the MOI of 0.062 TU/cell. At the indicated time points p.t., cells were processed for MTT assays to calculate cell metabolic activity. Data are expressed as a percentage of those obtained for cells transduced with the control lentivirus pLL3.7/U6-shScrambled. Data are the mean + standard error of the mean of three independent experiments performed in triplicate. (**C**) 2.1 × 10^4^ Huh7.5 cells/cm^2^ were seeded in 12-well plates and, 24 h later, transduced at MOI of 1.25 TU/cell for each lentiviral vector targeting the indicated sequence. 72 h p.t., cells were lysed and processed for shRNA quantification as described in the Materials and Methods section. Data shown are the expression of the specific siRNA from the indicated lentiviral vectors relative to that achieved after transduction with the single shRNA lentiviral vector. Green bars: shRNA located in the first position of the promoter array. Yellow bars: shRNA located in the second position of the promoter array. Blue bars: shRNA located in the third position of the promoter array. Red bars: lhRNAs expressed under transcriptional control of the U6 promoter. Data are the mean ± standard error of the mean relative to three independent experiments.

In order to generate novel combinatorial platforms able to interfere with HCV replication, first of all, we consulted previous literature data and online HCV resources to select 4 different shRNAs already known to efficiently block the viral life cycle: the IRES specific HCV353 and HCV321 siRNAs as well as siRNAs targeting the PI4KII and CypA host factors [18,20,32]. Furthermore, a scrambled control was also designed by adopting an ad hoc software tool (https://www.genscript.com/tools/create-scrambled-sequence, accessed 13^th^ October 2018). Expression of the different shRNA cassettes was placed under control of the human U6 small nuclear RNA, H1, and 7SK pol-III promoters. Next, we inserted the pol-III/shRNA cassettes in the context of pLentiLox3.7 (pLL3.7) self-inactivating lentiviral vector [48] that also expresses the EGFP reporter protein (Figure 1A and supplementary Figure S1). The anti-HCV molecules were either cloned as single transcriptional units (U6/shRNA) or as triple cassette arrays, by adopting all the selected pol-III promoters in different combinations, following the same cloning strategy previously described [33]. The U6 promoter was also employed to express lhRNAs. Specifically, two vectors were generated, named pLL3.7/U6-lhHCV321-PI4KIIIα and pLL3.7/U6-lhHCV321-CypA, both bearing two RNAi molecules under the transcriptional control of the U6 promoter. The list of generated vectors is reported in Figure 1A and supplementary Figure S1. Next, pseudotyped recombinant vectors were generated and quantified as previously described [33], and the same amount was employed to transduce the hepatocyte-derived cellular carcinoma cell line Huh7.5 at MOI of 0.062 TU/cell. Transduction efficiency ranged from 30 to 60% for all recombinant lentiviral particles. None of the selected lentiviral vectors showed a significant cytopathic effect on Huh7.5 cells with respect to the scrambled-transduced control (set as 100%), at least at 48 and 72 h post-transduction (p.t.), as shown by the effect of transduction and anti-HCV shRNA expression on cell viability/growth by adopting an MTT assay (Figure 1B).

Importantly, by adopting the Chen’s stem–loop RT-PCR method [46], we were able to show that all shRNAs are expressed in Huh7.5 cells already at 72 h p.t. for all the generated combinatorial platforms (Figure 1C). Of note, our results indicated that, overall, the U6 promoter placed in the first position of the expression cassette array leads to higher shRNA expression levels than the H1 or 7SK promoters, placed downstream. Furthermore, lhRNAs efficiently expressed both shRNA sequences, thus supporting their implementation for strategies aimed at simultaneously expressing two shRNAs.

### 3.2. Establishment of a Novel HCV Replicon Double Reporter Cell Line 

**Figure 2:**
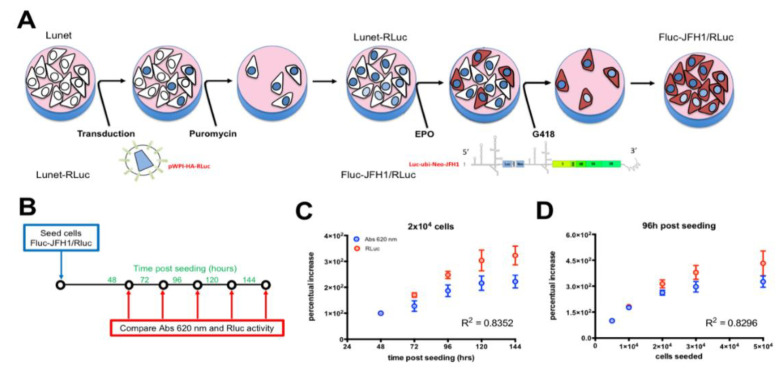
Generation of an HCV subgenomic double reporter replicon cell line allows accurate quantification of cell number. (**A**) Lunet cells were transduced with pWPI-HA-RLuc, mediating expression of the HA-RLuc reporter under control of the EF1α promoter. Transduced cells were selected using Puromycin to generate Lunet-RLuc cells, allowing to easily monitor cell number and viability by firefly luciferase assays. Subsequently cells were electroporated with in vitro transcribed RNA from plasmid pFKI389Luc-ubi-neo/NS3-3′_dg_JFH. Cells stably replicating HCV subgenomic RNA were selected using G418 (750 µg/mL) to generate FLuc-JFH1/RLuc cells, allowing to simultaneously monitor cell number and viral replication by dual luciferase assays. (**B**). Different amounts of FLuc-JFH1/RLuc cells were seeded either in 96- (for MTT assays) or 24- (for RLuc assays) well plates and incubated for the indicated time points before being processed as described in the Materials and Methods section. (**C**) Measurements relative to 2 × 10^4^ cells seeded/cm^2^, cultured for the indicated time, are expressed as the percentage of the signals obtained 48 h post-seeding. The R^2^ relative to the linear regression between Abs at 620 nm (blue circles) and RLuc values (red circles) is shown. Data are mean + standard error of the mean relative to three independent experiments. (**D**) Data relative to the indicated number of cells seeded/cm^2^ and cultured for 96 h, are expressed as the percentage of the signals obtained for 5 × 10^3^ cells seeded/cm^2^. The R^2^ relative to the linear regression between Abs 620 nm (blue circles) and RLuc values (red circles) is shown. Data are mean ± standard error of the mean relative to three independent experiments.

In order to (i) allow easy quantification of the levels of HCV replication in cell culture and (ii) simultaneously normalize for well-to-well variations in cell number linked to variance in cell seeding, as well as effects on cell growth and viability due to drug treatments, transfection, and lentiviral transduction, we developed a new HCV replicon double-reporter cell line (Figure 2A). To this end, we firstly transduced Huh7-Lunet, a subgenomic HCV-cured cell line that allows high levels replication of HCV RNA [40], with bicistronic lentiviral particles mediating the expression of a HA-RLuc fusion protein under the control of the EF1α promoter. Selection with puromycin allowed to select a polyclonal population stably expressing RLuc (Lunet-RLuc). Subsequently, Lunet-RLuc cells were electroporated with in vitro transcripts from plasmid pFKI389Luc-ubi-neo/NS3-3′_dg_JFH, thus introducing a bicistronic subgenomic FLuc-expressing reporter HCV replicon, conferring resistance to G418 upon RNA replication. Prolonged incubation of such cells with G418 resulted in selection of the FLuc-JFH1/RLuc replicon cell line. We tested the utility of this cell line to quantify the cell number by MTT assay and RLuc activity. Different amounts of FLuc-JFH1/RLuc cells were seeded either in 96 (for MTT assays) or 24 (for RLuc assays) well plates and cultured for different times, before being processed as described in the Materials and Methods section for evaluation of cell growth through MTT or RLuc activity assays (Figure 2B). Our results show that the data obtained from MTT and RLuc assays were highly comparable (Supplementary Figure S2). Indeed, the percentual increase of Abs at 620 nm measured by MTT assays well correlated with the percentual increase measured by RLuc assays when the same cell number was monitored for up to 144 h post-seeding (Figure 2C) or when different cell numbers were seeded and analyzed at the same time post-seeding (Figure 2D). This finding implies that the RLuc (cell viability) and FLuc (HCV replication) values can be obtained in a single assay, thus simplifying sample handling, as well as data acquisition and analysis (Figure 2C,D).

**Figure 3:**
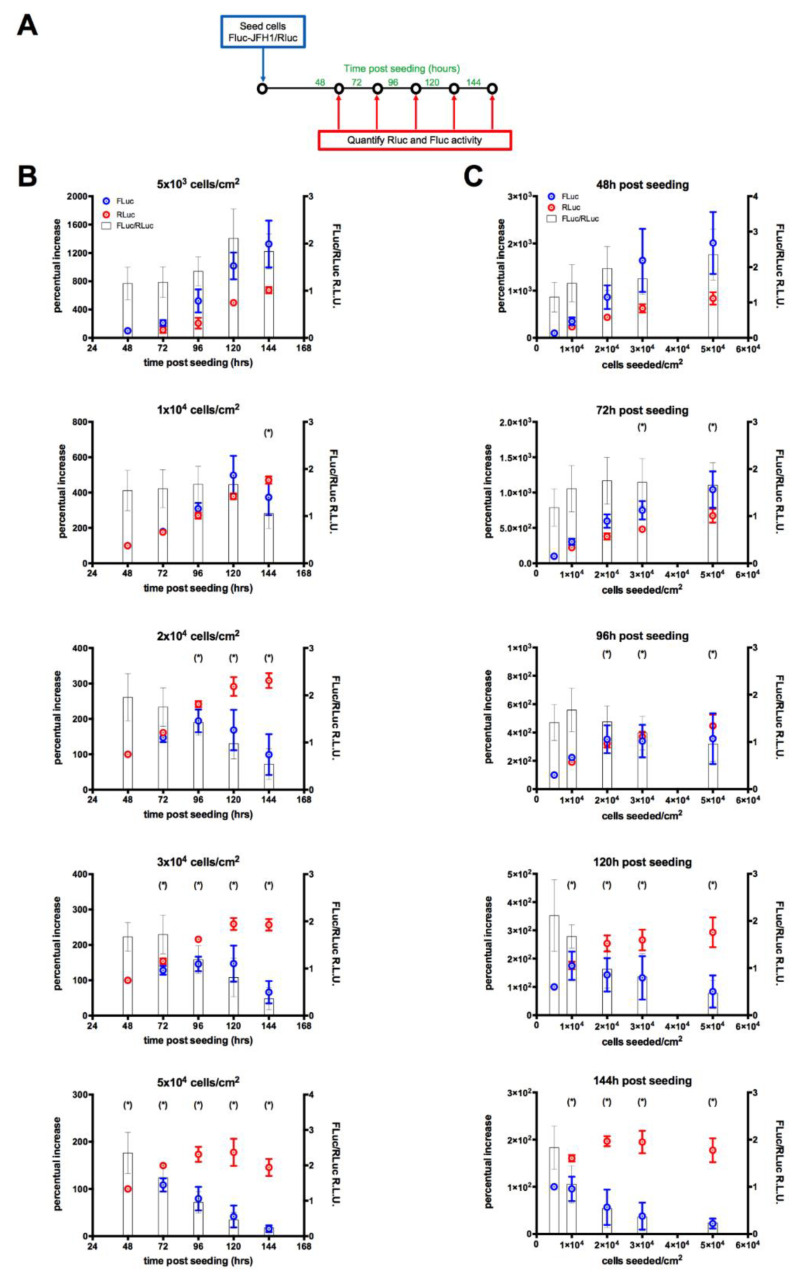
Quantification of cell number and viral replication of a HCV double reporter subgenomic replicon cell line. (**A**). Different amounts of FLuc-JFH1/RLuc cells were seeded in 24-well plates and incubated for the indicated time points before being lysed and processed for luminometric detection of RLuc and FLuc signals, allowing to calculate the relative HCV replication as reported by the FLuc/RLuc ratio. (**B**) FLuc (blue circles) and RLuc (red circles) were expressed as the percentage of the signals obtained for the indicated different times post-seeding and number of cell seeded, relative to 48 h post-seeding, whereas the FLuc/RLuc ratios relative to each condition are shown as white bars. (**C**). FLuc (blue circles) and RLuc (red circles) were expressed as the percentage of the signals obtained at the indicated time point post-seeding and number of cell seeded, relative to 5 × 10^3^ cells cm^2^/seeded, whereas the FLuc/RLuc ratios relative to each condition are shown as white bars. Data are mean ± standard error of the mean relative to data repeated in three independent experiments. * = cells overconfluent.

Next, we tested the suitability of such a replicon cell line for quantitatively correlating viral replication and cell growth by measuring the FLuc/RLuc activity. To this end, different amounts of FLuc-JFH1/RLuc cells were seeded in 24-well plates and lysed for quantification of viral replication (FLuc) and cell number (RLuc) at different time points post-seeding (Figure 3A). This allowed us to assess the effect of cell confluency, evaluated by light microscopy, on HCV replication. Our results indicated an inhibition of HCV RNA replication when cells were overconfluent (Figure 3B,C). This was even more evident when the FLuc/RLuc ratio was used as a marker of viral RNA replication normalized for the cell count, and consistent with previously published data [49].

### 3.3. Monitoring HCV Replication Inhibition with the FLuc-JFH1/RLuc Cell Line

**Figure 4:**
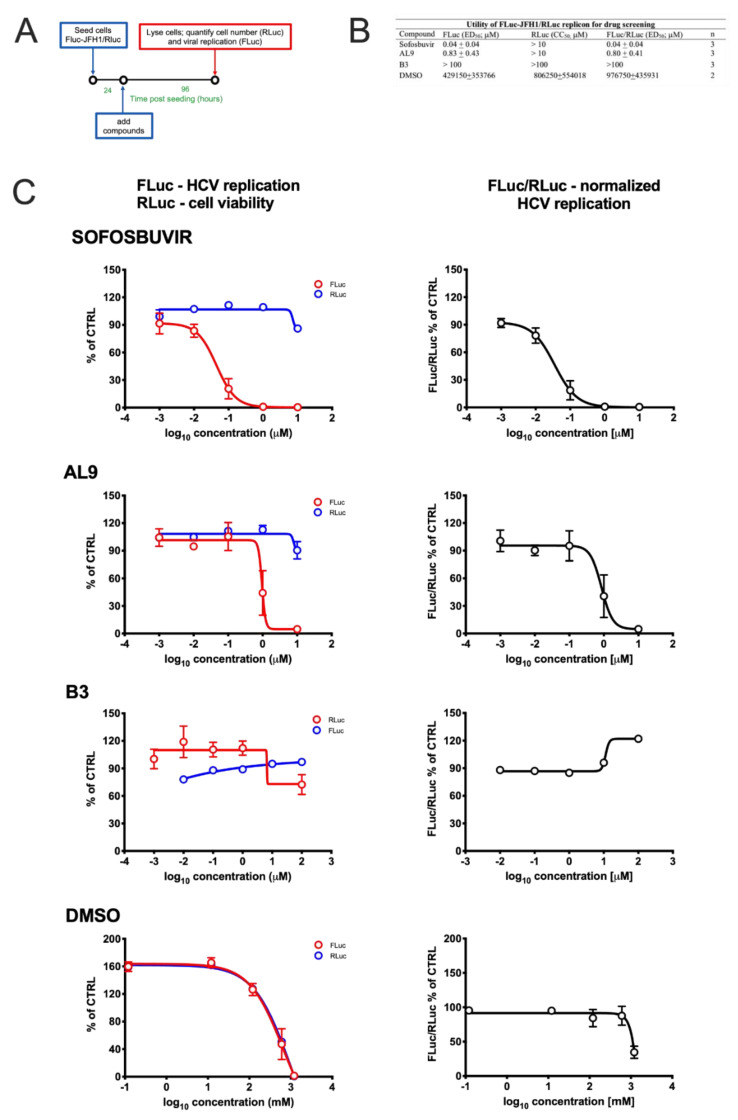
Effect of different compounds on HCV replication and cell viability. (**A**) FLuc-JFH1/RLuc cells were seeded in 24-well plates (2.5 × 10^4^ cells/cm^2^); 24 h later, cells were treated with increasing concentrations of the indicated compounds; 72 h post-treatment, cells were lysed and processed for luciferase assays for the quantification of viral RNA replication (FLuc) and cell number/viability (RLuc), as described in the Materials and Methods section. (**B**) Curves such as those shown in (**C**) were analysed as described in the Materials and Methods section in order to calculate the effective dose 50 (ED_50_; FLuc), cell culture 50 (CC_50_; RLuc) and normalized ED_50_ (FLuc/RLuc), relative to the indicated compounds. Data shown are the mean + standard deviation of the mean relative to the indicated number of independent experiments (*n*). (**C**) The FLuc (left panels, blue lines) and RLuc (left panels, red lines) activities, as well as the FLuc/RLuc ratio (right panels) relative to each condition were expressed as a percentage of the vehicle treated cells and representative curves relative to the indicated treatments are shown.

To assess the utility of our double replicon cell line as a tool to quantify the inhibition of HCV replication by novel therapeutic interventions, we started by testing the effect of well-known HCV inhibitors in cell culture. To this end, cells were seeded in 24-well plates and, 24 h later, treated with increasing concentrations of either the nucleoside NS5B inhibitor Sofosbuvir [50], or AL-9, an inhibitor of the host cell kinase PI4KIIIα, essential for HCV replication [29]. As controls B3, a compound active agent targeting Human Cytomegalovirus (HCMV; Alvisi et al., unpublished) or DMSO, an agent endowed with cytotoxic properties at high concentrations, were also included (Figure 4A). As expected, both Sofosbuvir and AL-9 inhibited HCV replication with ED_50_ values comparable to those described in the literature (40 ± 40 and 800 ± 410 nM, respectively), without observable cytotoxic effects (Figure 4B). On the other hand, no effect on HCV replication and cell viability was observed after incubation with the control molecule B3, whereas a strong inhibition of HCV replication was observed upon treatment with high concentrations of DMSO. However, such inhibition corresponded to a similar decrease in the cell viability/number, indicating a non-specific, cytotoxic-mediated effect. Therefore, the developed FLuc-JFH1/RLuc cell line allowed easy discrimination between specific and unspecific inhibition against HCV replication (Figure 4C). To verify if FLuc activity correlated with HCV replication we assessed the effect of Sofosbuvir on both FLuc activity, by means of luciferase assays, and HCV protein expression, by Western blotting. Our results showed that FLuc activity well correlated with NS5A expression, further confirming the utility of our double reporter cell line (Supplementary Figure S3).

### 3.4. Lentiviral Transduction of the FLuc-JFH1/RLuc Replicon Cell Line Results in MOI-Dependent Inhibition of Viral Replication

**Figure 5:**
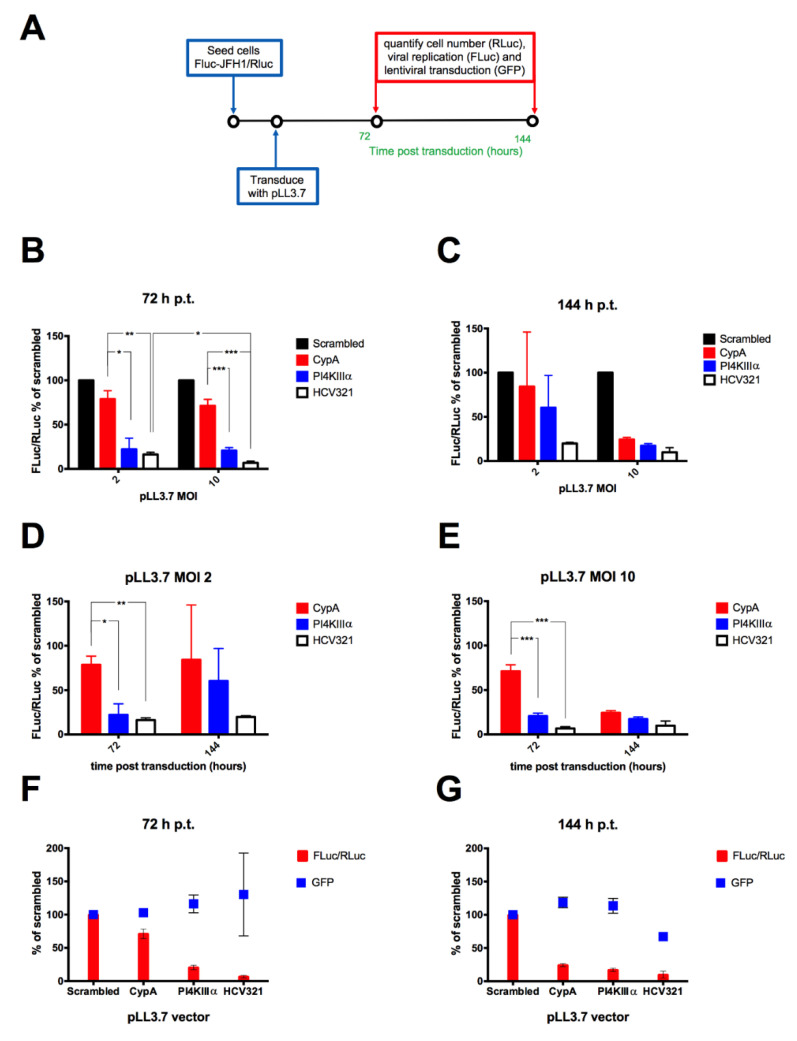
MOI-dependent inhibition of HCV replication as mediated by single miRNA-expressing pLL3.7 vectors. (**A**) 2 × 10^4^ cells/cm^2^ and 1 × 10^4^ cells/cm^2^ of FLuc-JFH1/RLuc cells were seeded in 24-well plates, cultured at 37 °C in a humidified incubator for further 24 h, and transduced at a MOI of 2 or of 10 TU/cell with pLL3.7-derived lentiviral particles encoding single shRNAs targeting the indicated sequences for 72 and 144 h, respectively. At the indicated time points p.t., cells were lysed and processed for fluorimetric detection of transduction efficiency (GFP) and luminometric detection of cell number (RLuc) and HCV replication (FLuc), allowing to calculate the relative HCV replication indicated by the FLuc/RLuc ratio. The FLuc/RLuc ratio relative to cells transduced with the indicated lentiviruses is shown at 72 (**B**) and 144 (**C**) h p.t., and for MOI of 2 (**D**) and MOI of 10 (**E**), expressed as a percentage of the mean values obtained for cells transduced with pLL3.7 encoding for a non-targeting shRNA (Scrambled). The latter, relative to cells transduced with a MOI of 10 is also compared to the GFP/RLuc ratio expressed as percentage of mean values obtained for cells transduced with pLL3.7 encoding for a non-targeting shRNA (Scrambled) at 72 (**F**) and 144 (**G**) h p.t. Data are the mean + standard error of the mean relative to three independent experiments. * = *p* ≤ 0.05; ** = *p* ≤ 0.005; *** = *p* ≤ 0.0005.

The new cellular model was used to evaluate the effect of lentiviral-mediated shRNA expression on HCV replication using pLL3.7 lentiviral particles encoding for individual anti-HCV shRNAs either targeting the HCV genome (HCV321) or essential host factors, such as PI4KIIIα and CypA. A scrambled control was also included. To this end, cells were seeded in 24-well plates and 24 h later transduced with lentiviral particles at a MOI of 2 and 10 TU/cell, before being processed at 72 and 144 h p.t. for quantification of cell number (RLuc), viral replication (FLuc) and transduction efficiency (GFP), in order to normalize viral replication (FLuc/RLuc) and transduction efficiency (GFP/RLuc) for the cell number (Figure 5A). All tested recombinant lentiviral particles showed antiviral effects and inhibited viral replication with variable efficacy and kinetics (Figure 5B–E). The strongest anti-viral activity was observed after transduction with pLL3.7/U6-shHCV321. For all lentiviral vectors tested, transduction at a MOI of 10 TU/cell resulted in a stronger antiviral effect (Figure 5B,C). This phenomenon was more evident at 144 h p.t., suggesting a more sustained inhibition of viral replication over time when transducing cells at an higher MOI (Figure 5D,E). Importantly, differences observed in viral replication inhibition did not depend on variability in cell transduction, as assessed by quantification of GFP fluorescence (Figure 5F,G). Statistical analysis indicated that at 72 p.t. cells transduced with pLL3.7/U6-CypA at a MOI of 2 and 10 TU/cell reduced viral replication to a significantly lesser extent than transduction with either pLL3.7/U6-PI4KIIIα (*p* = 0.0112 and 0.0007, respectively) or pLL3.7/U6-shHCV321 (*p* = 0.0070 and 0.0002, respectively). However, no statistically significant difference was observed between cells transduced with the different lentiviral particles targeting HCV replication at 144 p.t.

### 3.5. A Combinatorial Platform to Efficiently Inhibit HCV Replication

**Figure 6:**
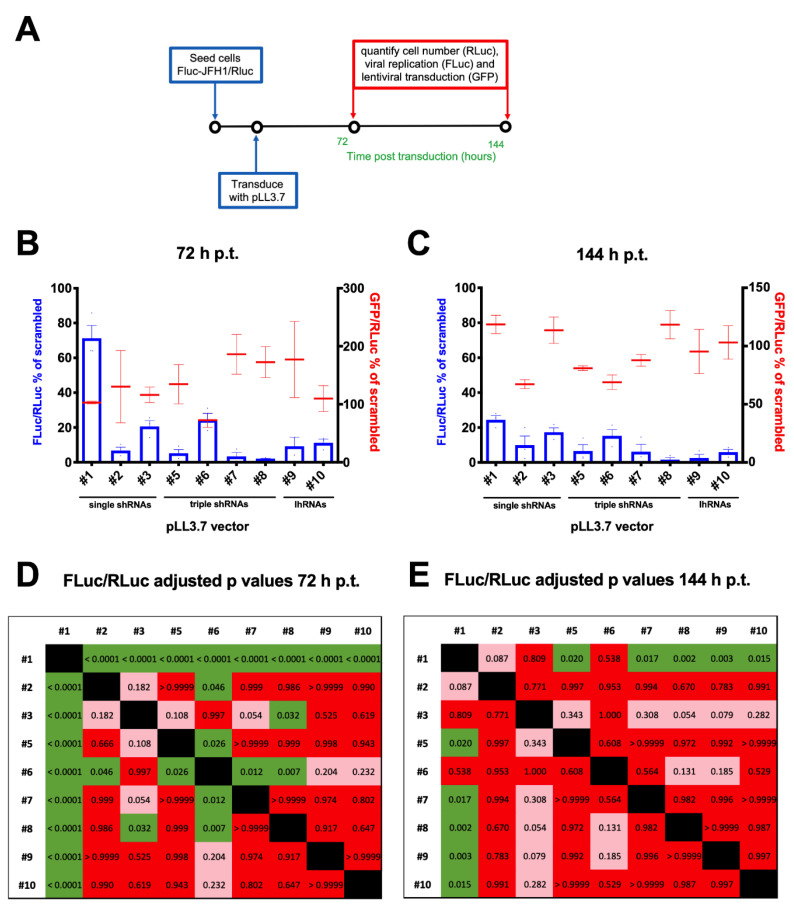
Inhibition of HCV replication as mediated by the combination of miRNA-expressing pLL3.7 vectors. (**A**) 2 × 10^4^ cells/cm^2^ and 1 × 10^4^ cells/cm^2^ of FLuc-JFH1/RLuc cells were seeded in 24-well plates, and after 24 h were transduced with different lentiviral vectors encoding for a combination of shRNAs/lhRNAs targeting the sequences described in the Material and Methods for 72 and 144 h p.t., respectively. At the indicated time points p.t., cells were lysed and processed for fluorimetric detection of transduction efficiency (GFP) and luminometric detection of cell number (RLuc) and HCV replication (FLuc). The FLuc/RLuc (blue columns) and GFP/RLuc (red bars) ratios relative to cells transduced with the indicated pLL3.7 shRNA/lhRNA lentiviruses at 72 (**B**) and 144 (**C**) h p.t., are expressed as a percentage of the mean values obtained for cells transduced with pLL3.7 encoding for a non-targeting shRNA (Scrambled). Data are the mean + standard error of the mean relative to three independent experiments. Adjusted *p* values from the Turkey multiple-comparison post-test are reported for the FLuc/RLuc ratios relative to cells transduced with each lentiviral particle at 72 (**D**) and 144 (**E**) h p.t. Green: *p* value < 0.05; pink: 0.05 < *p* value < 0.5; red: *p* value > 0.5; black: no *p* value calculated. Lentiviral vectors were as follows: #1, pLL3.7/U6-shCypA; #2, pLL3.7/U6-shHCV321; #3, pLL3.7/U6-shPI4KIIIα; #4, pLL3.7/U6-shScrambled;#5, pLL3.7/U6-shHCV321-H1-shCyp-7SK-shHCV353;#6, pLL3.7/U6-shPI4KIIIα-7SK-shHCV321-H1-shCypA; #7, pLL3.7/U6-shHCV321-7SK-shPI4KIIIα-H1-shHCV353; #8, pLL3.7/U6-shHCV321-7SK-shPI4KIIIα-H1-shCypA; #9, pLL3.7/U6-lhHCV321-CypA; #10, pLL3.7/U6-lhHCV-PI4KIIIα.

Since inhibition of viral replication with a single shRNA-expressing cassette might promote the selection of resistant escape viral mutants, we tested the ability of 6 combinatorial lentiviral particles simultaneously expressing different shRNAs, to specifically inhibit HCV replication. To this end, the FLuc-JFH1/RLuc replicon cell line was transduced with 10 different lentiviral particles at a MOI of 10 and the effect of viral replication was assessed at 72 and 144 h p.t. (Figure 6A). RLuc (cell number), FLuc (HCV replication) and GFP (transduction efficiency) activities were measured, in order to normalize viral replication (FLuc/RLuc) and transduction efficiency (GFP/RLuc) for the cell number (Figure 6B,C). Statistical analysis indicated that transduction with the 10 different lentiviral particles affected viral replication to a statistically different extent, both a 72 and 144 p.t. (*p* values < 0.0001 and 0.0012, respectively), in the absence of statistically significant differences in the transduction efficiency. Both at 72 (Figure 6B) and 144 h p.t. (Figure 6C), the combinatorial vectors showed inhibitory effects comparable or higher to those achieved by the single shRNA-expressing lentiviral vectors. Importantly, lentiviral vector #1 (pLL3.7/U6-shCypA) resulted in the lowest inhibition of HCV replication, followed by vectors #3 (pLL3.7/U6-shPI4KIIIα) and #6 (pLL3.7/U6-shPI4KIIIα-7SK-shHCV321-H1-shCypA). Overall, the strongest inhibition was observed 144 h p.t. after transduction with lentiviral vector #8 (pLL3.7/U6-shHCV321-7SK-shPI4KIIIα-H1-shCypA). Importantly, the differences observed in viral replication inhibition did not depend on the variability in cell transduction, as assessed by quantification of GFP fluorescence after normalization for the cell number using the RLuc activity (Figure 6B,C; red bars). Post-test ANOVA analysis of the effect of transduction with the different lentiviral particles revealed that at 72 h p.t., vector #1 was significantly less active than all other vectors (Figure 6D). In addition, vectors #2 (pLL3.7/U6-shHCV321), #5 (pLL3.7/U6-shHCV321-H1-shCyp-7SK-shHCV353), #7 (pLL3.7/U6-shHCV321-7SK-shPI4KIIIα-H1-shHCV353) and #8 (pLL3.7/U6-shHCV321-7SK-shPI4KIIIα-H1-shHCV353) significantly inhibited viral replication also more than vector #6, while only vector #8 significantly inhibited viral replication more than vector #3 (pLL3.7/U6-shPI4KIIIα) (see Figure 6D for adjusted *p* values). At 144 h p.t., however, only transduction with combinatorial vectors inhibited viral replication to significantly higher levels as compared to vector #1, with vector #8 also almost reaching significance (*p* = 0.054) as compared to vector #3 (see Figure 6E for adjusted *p* values). These data suggest that lentiviral particles expressing shRNAs and lhRNAs with our combinatorial strategy can inhibit HCV replication at least as efficiently as single shRNA expressing vectors.

Overall, these results highlight the possibility of adopting a combinatorial approach for robust and long-term inhibition of HCV replication.

## 4. Discussion

Although the introduction of direct acting antivirals (DAAs) has significantly improved HCV therapy, this virus still infects millions of people worldwide, representing one of the major causes of liver disease associated with significant morbidity and mortality [51]. HCV patients were initially treated with a combination of interferon and ribavirin, a therapy characterized by important side effect and low efficacy [52]. In 2011, DAAs were introduced for the treatment of people chronically infected with HCV. This combinatorial therapy can lead to sustained virologic response rates over 90% for most viral genotypes [53]. Despite this success, the significant costs of the DAA therapy along with different severe side effects that result in lack of patient compliance before viral clearance are still unsolved issues [54]. Furthermore, it has been demonstrated that HCV variants resistant to DAAs might be present even before the initiation of treatment [55,56]. Due to its biological features, HCV can be easily targeted by small RNAs able to interfere with viral genomic sequences essential for replication. In this context, the NTR mapping at the 5′ of the genome, being highly conserved among different HCV isolates, represents an interesting target for the design of new therapies for the treatment of hepatitis C based on RNAi. Furthermore, several studies have clearly showed that, as in the case of HIV, the simultaneously expression of more than one RNAi sequences is more efficient in blocking viral replication as well as in preventing escape mutations [57,58]. In this context, the simultaneous targeting of viral and host factors involved in viral replication represents a successful strategy in terms of both antiviral effect and prevention of viral mutant selection [5,6,7,8,9,10,11,12].

Building on previous published work on RNAi strategies aimed at blocking HCV replication/infection and on our experience with HIV-1 [33], we decided to develop an array of lentiviral vectors expressing different combination of shRNAs targeting both the HCV genome (the IRES sequence) and host factors involved in viral life cycle (PI4KIIIα and CypA). Lentiviral vectors expressing single shRNAs and a Scrambled sequence were also developed as controls. With the aim of selecting the best performing platforms, we set up a series of experiments to test the generated recombinant lentiviral particles in terms of (i) efficiency in transferring and expressing the selected therapeutic sequences into a relevant cell line; (ii) cytotoxicity towards the same cell type; and (iii) their antiviral activity.

Firstly, we selected Huh7-derived cells as experimental setting [40]. Such cell lines were adopted since they represent well characterized hepatocyte-derived cellular carcinoma cell lines, extensively used in HCV research and allowing high levels of viral RNA replication [59].

**Figure 7:**
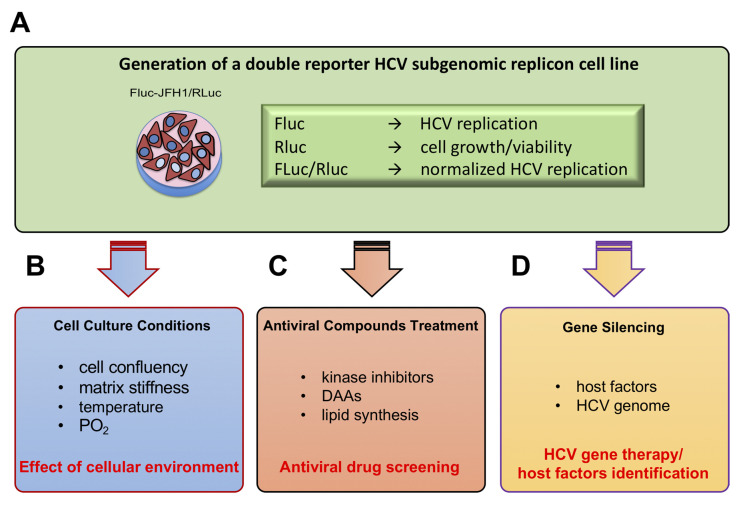
Schematic representation of the study experimental set up. (**A**) We established a double reporter HCV replicon cell line allowing simultaneous luminometric quantification of HCV genotype 2a JFH1 replication (FLuc) and cell proliferation/viability (RLuc). Such cell line can be used for several applications including: testing the effect of cell culture conditions on HCV replication (**B**), antiviral compounds identification/screening (**C**), or gene silencing experiments aimed at identifying new host factors, as well as optimization of conditions for therapeutic gene silencing (**D**).

We also developed and characterized a novel JFH1-based HCV replicon double reporter cell line (FLuc-JFH1/RLuc), as briefly summarized in Figure 7, which enables for normalization for well-to-well variations in cell number (Figure 7A and Figure 3) and is thus amenable to study the effects of culture conditions, such as cell confluency, on HCV RNA replication (Figure 7B and Figure 3).

The FLuc-JFH1/RLuc replicon cell line also allows discrimination between the antiviral effect due to treatment with specific inhibitors of HCV replication, such as the NS5B inhibitor and the PI4KIIIα inhibitor AL-9, and inhibition of viral replication due to cell toxicity, such as in the case of cell treatment with high concentrations of DMSO (Figure 7C and Figure 4). Importantly, we also showed that FLuc activity directly correlated with HCV protein expression as assessed by Western blotting (Supplementary Figure S3), in agreement with earlier reports [60].

Using this cell line, we demonstrated that transduction with recombinant lentiviral particles, pseudotyped with the vesicular stomatitis virus (VSV) G envelope protein expressing a single shRNA cassette, inhibited HCV replication to different extents. Normalization for cell number and GFP expression allowed to exclude that such differences were due to variability in cell seeding, viability, and transduction, but rather relied upon the target sequence. Indeed, our results clearly indicated that the strongest antiviral activity was observed upon expression of shRNA directly targeting the viral genome, such as in the case of lentiviral vector #2, pLL3.7/U6-shHCV321 (Figure 7D and Figure 5).

The observation that, with the exception of vector #6, the double and triple-cassette expressing vectors generated in this study inhibited HCV replication to a similar extent as pLL3.7/U6-shHCV321, is of interest in the context of a possible combinatorial strategy targeting multiple viral and cellular genes to minimize the selection of resistant viral variants [13,14,15,16]. Furthermore, we observed GFP expression for more than 1 month upon transduction of Huh7.5 cells with the scrambled-expressing vector [61]). Thus, these combinatorial platforms appear to be particularly attractive to simultaneously express two anti-HCV molecules, one targeting viral conserved sequences and one interfering with a cellular factor, a strategy already proved to be effective [58]. However, quantitative analysis of shRNA expression levels revealed that while transduction with vector #9, pLL3.7/U6-lhHCV321-CypA and vector #10, pLL3.7/U6-lhHCV321-PI4KIIIα, which express lhRNAs, led to an efficient production of both RNAi molecules (Figure 1C; red bars), in the triple cassette vectors the antiviral molecules in second and third position are less efficiently produced (Figure 1C; yellow and blue bars, respectively). Importantly, since the pol-III promoter we adopted in first position is always U6, our data do not allow to conclude whether the expression differences depend on the promoter or on the cassette position. Nonetheless, this finding seems to be in contrast with previous published work that found similar activities for the three promoters in expressing anti-HIV shRNAs [62]. Therefore, our result suggests that lhRNAs expression mediated by a single promoter appears superior as compared to the expression of individual shRNAs by multiple promoters. In the latter scenario, it might be worth placing in the downstream transcription unit shRNA sequences targeting host factors for which partial ablation is sufficient to severely impair virus replication, such as PI4KIIIα [63].

In conclusion, we have developed an array of lentiviral vectors simultaneously expressing multiples anti-HCV shRNAs that could find application in the development of innovative therapies, as well as in the study of virus biology and pathogenesis of infection. Furthermore, we developed an HCV replicon double reporter cell line that could be adopted for testing new therapeutic interventions/molecules aimed at interfering with HCV replication cycle.

## Supplementary Materials

The following are available online at https://www.mdpi.com/1999-4915/12/9/1044/s1, Figure S1: Schematic representation of the pLL3.7—derived third-generation self-inactivating lentiviral vectors generated in this study, Figure S2: Generation of an HCV subgenomic double reporter replicon cell line allows accurate quantification of cell number, Figure S3: Comparison between FLuc and Western blot assays for assessment of HCV replication.
