# Supplementary material for: Generation of Combinatorial Lentiviral Vectors Expressing Multiple Anti-Hepatitis C Virus shRNAs and Their Validation on a Novel HCV Replicon Double Reporter Cell Line
Source: Viruses. 2020 Sep 18;12(9):1044. doi: 10.3390/v12091044 (PMC7551853; doi:10.3390/v12091044)
Supplement: Supplementary file 1 [file viruses-12-01044-s001.pdf]

viruses

MDPI

Supplementary Figures

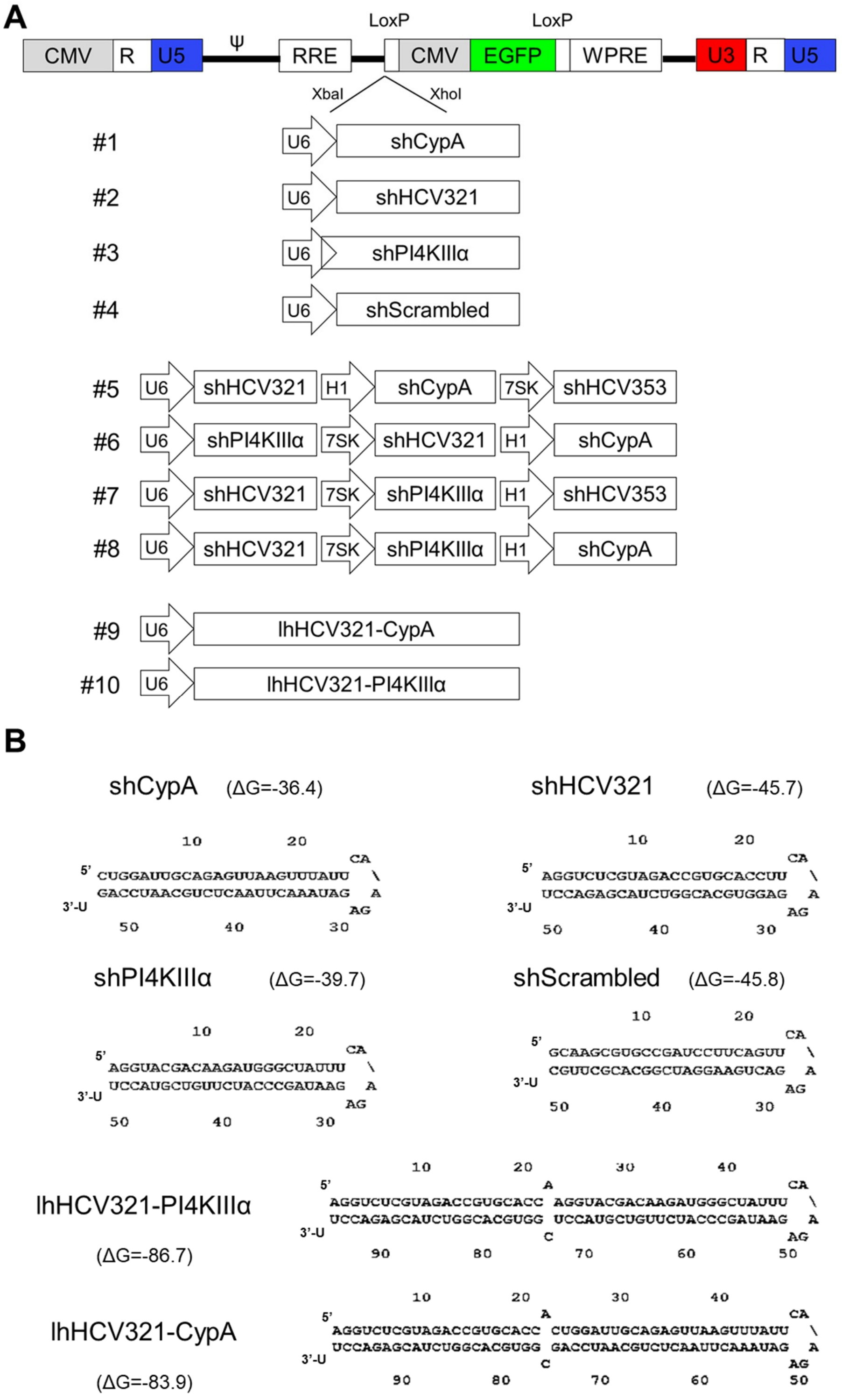

Supplementary Figure 1. (A) Schematic representation of the pLL3.7 – derived third-generation self-inactivating lentiviral vectors generated in this study. As indicated, different expression modules were cloned in the XhoI/XbaI restriction sites to allow expression of a number of different RNA molecules targeting HCV replication (*white boxes*) under the control of either U6, 7SK or H1 promoters (*white arrows*). (B) Secondary structure and thermodynamic stability (ΔG) for each hairpin molecule predicted using the Mfold web server as described in the Materials and Methods section.

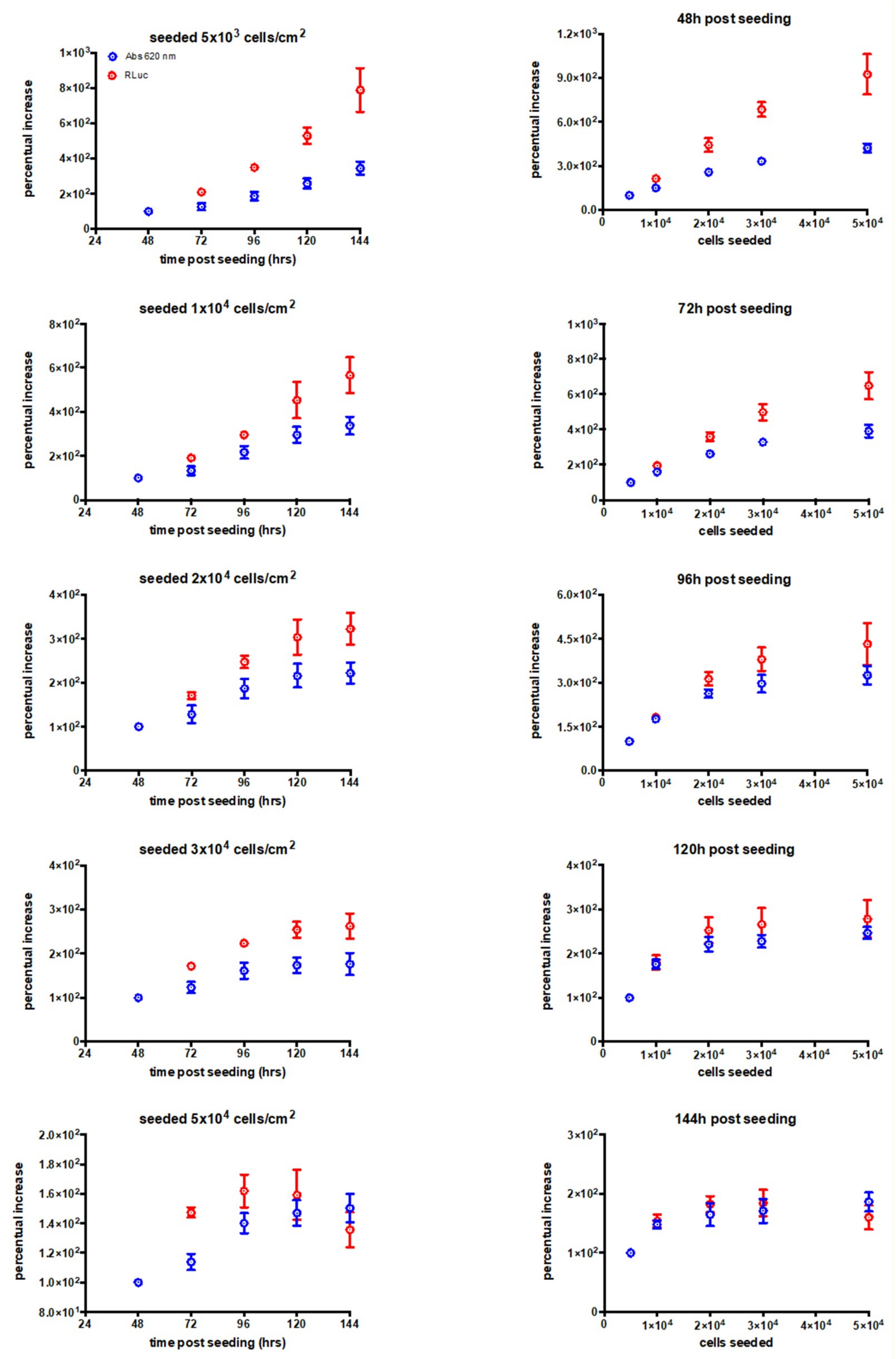

Supplementary Figure 2. Generation of an HCV subgenomic double reporter replicon cell line allows accurate quantification of cell number. Different amounts of FLuc-JFH1/RLuc cells were seeded in 24-well plates and incubated for the indicated time points before being lysed and processed for luminometric detection of RLuc and FLuc signal, allowing to calculate the relative HCV replication indicated by the FLuc/RLuc ratio. Data (FLuc activity, *blue circles*; RLuc activity, *red circles*) were expressed as the percentage of the signals obtained for the indicated number of cell seeded, at different times post-seeding (*left graphs*), or as the percentage of the signals obtained at the indicated time point post-seeding (*right graphs*). Data are mean ± standard error of the mean relative to three independent experiments.

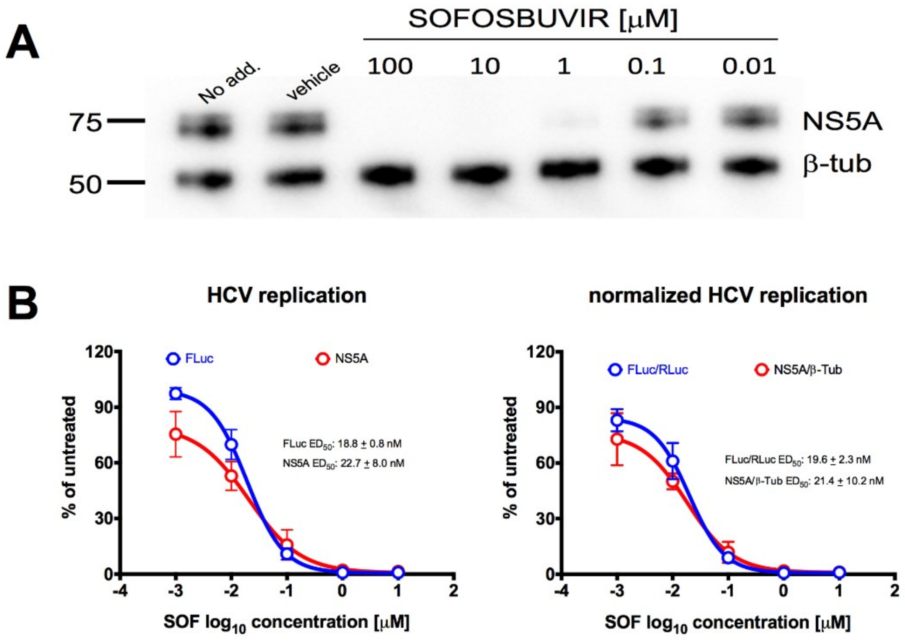

Supplementary Figure 3. Comparison between Fluc and Western blot assays for assessment of HCV replication. FLuc-JFH1/RLuc cells were seeded in 6-well plates (2.5x10^4 cells/cm^2) and 24 hours later were treated with increasing concentrations of Sofosbuvir in DMSO 0.1% (v/v). 72 hours later, cells were either washed and lysed in RIPA buffer with protease and phosphatase inhibitors and processed for SDS page/Western blotting or for luciferase assays as described in Material and Methods section. (A) A representative Western blotting experiment showing NS5A and β-tubulin specific bands. (B) The FLuc activity (*blue circles*) and NS5A expression (*red circles*) were quantified as described in the Materials and Methods section, expressed as a percentage of vehicle treated cells and plotted against the concentration of Sofosbuvir. (C) The FLuc/RLuc activity (*blue circles*) and NS5A/β-tubulin expression (*red circles*) were quantified as described in the Materials and Methods section, expressed as a percentage of vehicle treated cells and plotted against the concentration of Sofosbuvir.
